# Improving the quality of translation for patients with language barriers: Rebecca Newell

**DOI:** 10.1093/eurpub/ckac129.639

**Published:** 2022-10-25

**Authors:** R Newell, S George, M Patel, H Dawe, M Roelas, F Wedmore, M Morris

**Affiliations:** Acute Medicine, Barts Health NHS Trust, London, UK

## Abstract

**Background:**

Patients with language barriers have worse health outcomes. To promote equitable care, efforts must be made to ensure effective communication with patients, including providing professional translation. In a busy, overstretched health service this can be difficult to achieve. Language translators can reduce barriers, improve safety and ensure impartiality.

**Description of the Problem:**

Aims - To understand the quality of translation and explore interventions that could improve this in a busy diverse hospital in East London.

**Methods:**

A quality improvement project in the Medical Admissions Unit of the Royal London Hospital was completed over two years. Patients with language barriers were identified, methods of translation were analysed and interventions to improve access to independent translation were introduced.

**Results:**

Cycle 1- All medical admissions over a two-week period were analysed. 36 patients had documented evidence of a language barrier with 7 (19.4%) having independent professional translation. 20 (55.6%) patients had family members providing translation, for 4 patients limited English was used and previous documentation was used for 3 patients.

Cycle 2- Posters and information leaflets were disseminated regarding how to access translation services. There was no improvement in the types of translation used. Only 1 patient had impartial professional translation, out of 43 patients identified.

Cycle 3- Advocates (Bengali speaking professional translators) were made available on the ward twice a week. Results showed no significant improvement, with only 6 (10%) identified having impartial professional translation. However staff and patient feedback has been positive.

**Lessons:**

Impartial professional translation is essential in reducing barriers and delivering equitable health care. This can be difficult to achieve in busy healthcare services. Education alone is not enough. Good accessibility to translation services is paramount in promoting usage.

**Key messages:**

• Healthcare services often fall short of providing impartial professional translation.

• Education alone is not enough. Good accessibility to translation services is paramount in promoting usage.

